# Aberrant Neural Activity in Patients With Bipolar Depressive Disorder Distinguishing to the Unipolar Depressive Disorder: A Resting-State Functional Magnetic Resonance Imaging Study

**DOI:** 10.3389/fpsyt.2018.00238

**Published:** 2018-06-05

**Authors:** Meihui Qiu, Huifeng Zhang, David Mellor, Jun Shi, Chuangxin Wu, Yueqi Huang, Jianye Zhang, Ting Shen, Daihui Peng

**Affiliations:** ^1^Division of Mood Disorders, Shanghai Mental Health Center, Shanghai Jiao Tong University School of Medicine, Shanghai, China; ^2^School of Psychology, Faculty of Health, Deakin University, Melbourne, VIC, Australia; ^3^Institute of Biomedical Engineering, School of Communication and Information Engineering, Shanghai University, Shanghai, China; ^4^Department of Medical Imaging, Shanghai Mental Health Center, Shanghai Jiao Tong University School of Medicine, Shanghai, China; ^5^Department of Psychiatry, Shanghai Mental Health Center, Shanghai Jiao Tong University School of Medicine, Shanghai, China

**Keywords:** bipolar disorder, unipolar depressive disorder, fractional amplitude of low frequency fluctuation, resting-state, functional magnetic resonance imaging

## Abstract

This study aims to explore the intrinsic patterns of spontaneous activity of bipolar depression (BD) patients by analyzing the fractional amplitude of low frequency fluctuation (fALFF) that help differentiate BD from unipolar depressive disorder(UD). Twenty eight patients with BD, 47 patients with UD and 29 healthy controls were enrolled to receive the resting-state functional magnetic resonance imaging (rs-fMRI) scans. The group differences of fALFF values were calculated among three groups. In addition, the correlations between the clinical variables and mfALFF values were estimated. The brain regions with activation discrepancies among three groups are located in precuneus, the left middle temporal gyrus (MTG) and left inferior parietal lobe (IPL) and lingual gyrus. Compared with HC group, BD group shows decreased fALFF in precuneus, the left IPL and increased fALFF in lingual gyrus remarkably; UD group shows significantly decreased fALFF in precuneus, the left MTG and the left IPL. On the contrast of patients with UD, patients with BD have significantly increased fALFF value in the left precuneus, the left MGT and lingual gyrus. Furthermore, a negative correlation is found between the mfALFF values in precuneus and the scores of cognitive impairment factor in the UD group. The similar pattern of intrinsic activity in PCC suggests depressive state-dependent change. The aberrant patterns of intrinsic activity in precuneus, the IPL and lingual gyrus might be provide quantitative nodes that help to conduct further study for better distinguishing between BD and UD.

## Introduction

Bipolar disorder, characterized by abnormal mood, cognitive dysfunctions, changes of behaviors, and disturbed circadian rhythms, ranks the twelfth burdens of all diseases in terms of disability-adjusted of Life Years (1). The precise diagnosis and efficient treatment have generally been limited by the fact that the dynamic process of bipolar disorder is dominated by depression or elevated mood (mania or hypomania). The hypomania is even more difficult to recognized in its earlier onset (2). More importantly, most individuals with bipolar disorder (58–71%) have depressive onset polarity, which usually show some overlapping clinical manifestations with the UD, such as depressed mood, retardation of thinking, and cognitive impairment. A latest meta-analysis for prospective transition from UD to bipolar disorder, reported that almost a quarter of individuals with UD transitioned to bipolar disorder after more than 12 years follow-up (3). Furthermore, the misdiagnosis of bipolar disorder would result in the delay of treatment, exerting a heavy economic burden on patients' families and the society (4, 5). Hence, it is imperative to find appropriate methods to distinguish bipolar disorder patients from individuals with UD, which could help to minimize the impact of mixed diagnoses in early therapeutic intervention.

Unfortunately, the current diagnostic schema for bipolar disorder, which is still based on descriptive psychopathology rather than clearly elucidating the neuropathophysiology, has failed to identify BD precisely (6). The current advances in multi-modal neuroimaging studies of bipolar disorder have effectively found the alterations in both brain structure and function, including gray matter morphology (7, 8), white matter integrity (9), and local/global network connectivity (10, 11), which could provide reliable neurobiological markers for bipolar disorder. Importantly, considerable neuroimaging studies have suggested that compared with UD patients, individuals with BD had significantly abnormal patterns of subcortical and cortical activity (12–14). Therefore, considering intrinsic neural characteristics without task-specific neural function, resting-state functional magnetic resonance (rs-fMRI) can provide useful biomarkers (3, 15, 16), which investigate the basic activity of brain during awake and resting state based on the blood oxygenation level dependent (BOLD) signal (17). However, only few studies based on rs-fMRI data directly compared BD with UD. Liu et al revealed that significant alteration of connectivity models of default mode network (DMN) were found in bipolar depressive disorder compared with unipolar depression using the pLiNGAM algorithm (18). Two additional studies (19, 20) showed abnormal values of regional homogeneity (ReHo) in BD group compared with UD group. These functional neuroimaging studies estimated functional integrity of neural circuitries relevant to neuropathophysiological processes in bipolar disorder, which have provided us with holistic information of patterns within brain networks from the perspective of functional integration, by measuring regional activity, using blood-oxygen level dependent (BOLD) signal change, and functional connectivity, using techniques examining the extent of coupling of time series of activity between neural regions of interest. However, most of previous studies used seed-voxel analysis to explore the regional brain activity (21). Studies of the intrinsic functional dynamics based on global brain voxels between BD and UD groups are insufficient.

For these reasons, it's important to explore the intrinsic spontaneous brain activity measured by resting-state fMRI (RS-fMRI) in samples of depression (22), Individual differences in spontaneous brain activity may reflect distinct experiences and learning histories (23). Considering the widespread region features of BOLD-fMRI signal, Zou et al. firstly applied the fast Fourier Transform technique to process the low frequency (0.01-0.08 Hz) signal of the whole brain voxels (24). The low frequency signals were further standardized by full frequency amplitude (0–0.25 Hz), which were defined as the fractional amplitude of low-frequency fluctuations (fALFF) (24). The fALFF might reflect the features of spontaneous neural activity, which is related to the rate of regional glucose metabolism and reflecting regional brain activity abnormalities at baseline (25, 26), avoid the influences of physiological noise, and provide the information about the brain impairment (24). The fALFF has been applied to directly reveal the spontaneous activity of each brain region and to precisely demonstrate that the significant alteration of cortical intrinsic activity is existed in many psychiatric disorders, such as schizophrenia, UD and obsessive-compulsive disorder (27–29). Although some neuroimaging studies by fALFF analysis showed the alterations of spontaneous activities of cortical-limbic circuits in patients with UD (30, 31), no studies found the similar changes in patients with BD. Furthermore, the abnormality of spontaneous neuronal activity in patients with UD and BD has not been totally elucidated. Evidences obtained from existing studies using the fALFF approach are insufficient and inconsistent.

There were insufficient studies to evaluate the alterations of global regions in characteristics of spontaneous neural activity between patients with BD and patients with UD by fALFF value. Accordingly, in the present study, we aimed to investigate the abnormal regions in BD and UD by analyzing fALFF. We hypothesized that individuals with BD, compared to UD and HCs, would exhibit aberrant intrinsic activity by fALFF value based on rs-fMRI data, extrapolating a potential clue to distinguish patients with BD from population with depression.

## Materials and methods

### Participants

Twenty-eight patients with BD, 47 patients with UD were enrolled from out-patient departments in Shanghai Mental Health Center. Twenty-nine healthy volunteers were recruited from society by advertisement as well. Diagnosis of BD and UD were based on the Structured Clinical Interviews for Diagnostic and Statistical Manual fourth edition (DSM-IV), the 24-item Hamilton rating scale for depression (HAMD) >20 (32), and the Young Mania Rating Scale (YMRS) <7 (33). Exclusion criteria for all participants included meeting any other diagnosis criteria of current or past Axis I and Axis II psychiatric disorders of DSM-IV, current alcohol/substance dependence, or abuse (within 6 months of study), positive urinary toxicology screening at baseline, serious neurological or medical disorders, positive pregnancy test or lactation, a history of head trauma, and general contraindications to MRI, a history of electroconvulsive therapy. All participants were right-handed Han Chinese with more than 9 years of schooling. The study was approved by the Investigational Review Board (IRB00002733-Shanghai mental health center, China), and written informed consent was obtained from all subjects before inclusion in the study.

### Image acquisition

Brain imaging data were acquired using magnetic resonance scanning (Siemens 3.0T). High-resolution T1 images were acquired by the gradient recalled echo (GRE) sequence as the following parameters: repetition time (TR) = 2,300 ms, echo time (TE) = 2.96 ms, field of view (Fov) = 24 × 24 cm^2^, slice thickness = 1.0 mm, 192 slices, gap = 0.0 mm, voxel = 1.0 × 1.0 × 1.0 mm^3^, matrix = 240 × 256, scanning time = 9 min 14 s. Resting-state images were collected by echo planar imaging (EPI) sequence as the following parameters: TR/TE = 2,000/30 ms, Fov = 220 ^*^220 mm, slice thickness = 4.0 mm, 33 slices, gap = 0.6 mm, voxel = 3.4 × 3.4 × 4.0 mm^3^, 200 bolds with GRAPPA on. For each participant, the rs-fMRI scanning lasted for 6 min and 46 s. During the scanning, the participants were kept awake with eyes closed, body still, and their respiration regular.

### Functional imaging data preprocessing

All images were processed by Data Processing Assistant for Resting-State fMRI (DPARSF) based on Statistical Parametric Mapping (SPM8) (http://www.fil.ion.ucl.ac.uk/spm) and the toolbox for Data Processing & Analysis of Brain Imaging (DPABI, http://rfmri.org/DPABI). For each participant's data, the first 10 time points and the data with excessive head motion (shift >2.5 mm, degree of rotation >2.5°) were discarded to ensure the steady state. After realignment using a 6 degrees-of-freedom linear transformation without re-sampling, we co-registered the individual structural images (T1-weighted MPRAGE) to the mean functional image. Then the structural images were segmented into gray matter, white matter, and cerebrospinal fluid. Based on the Diffeomorphic Anatomical Registration Through Exponentiated Lie algebra (DARTEL) tool, these segmented images was used to compute transformation parameters that coregistered individual native space to Montreal Neurological Institute (MNI). The nuisance covariates, as the Friston 24-parameter model, were to regress out from the realigned data, including the six head motion parameters, the first time derivations, signals of the global brain, cerebrospinal fluid and white matter. The remaining images were standardized by MNI system and re-sampled to 3^*^3^*^3 mm3 voxels, and operated spatial smooth adopting 8 mm full width at half maximum (FWHM) so as to enhance the image signal-to-noise ratio. After preprocessing, 3 participants from UD group, 3 from BD group and 2 from HC group were respectively excluded with the standard of head motion.

### fALFF analysis

The fALFF was computed with Resting-State fMRI Data Analysis Toolkit (REST) (http://www.restfmri.net). After undergoing a band-pass filter (0.01–0.08 Hz) (34) and linear-trend removing, we calculated the power spectrum by transforming the time series of each voxel with a fast Fourier transform (FFT). A ratio of the low-frequency amplitude averaged (0.01–0.08 Hz) to the power spectrum of the entire frequency range (0–0.25 Hz) was computed at each voxel to obtain the fALFF (24). For standardization, the fALFF value was further divided by the global mean fALFF value within a group-based gray matter (GM) mask that was generated by the mean GM map (threshold = 0.15) of all subjects, and the mean fALFF (mfALFF) was obtained.

### Statistical analysis

Demographic and clinical data were analyzed using SPSS, version 19.0 (SPSS, Inc., Chicago). The differences of voxel-based fALFF among BD group, UD group, and HC groups were analyzed by the REST using one-way analysis of variance (ANOVA), with age, gender and education level as covariates. Significant differences were set at the threshold of voxel-wise *P* < 0.001 (uncorrected), with a cluster size >10 voxels. The mean fALFF values of brain regions with significant differences were extracted for further *post-hoc* analysis among three groups. Statistical significance for *post-hoc* analysis was set at *P* < 0.01. Then, we performed the Pearson correlation analyses between the mean fALFF values of the brain regions showing significant group differences and the clinical characteristic variant (e.g., total HAMD and HAMA scores). Notably, the scale of HAMD was broken into seven factors based on its Chinese version: anxiety/somatization, change of weight, cognitive dysfunction, atypical circadian rhythm, retardation, sleep disorder, and desperation (35). The threshold for statistical significance was set at *P* < 0.05.

## Results

### Demographics and clinical characteristics

The demographic and clinical data of 28 participants with BD (14 male/14 female, aged 19–45 years, mean ± SD: 31.79 ± 12.83), 47 participants with UD (20 male/27 female, aged 21–51 years, mean ± SD: 38.11±13.16), and 27 healthy controls (17 male/10 female, aged 21–43 years, mean ± SD: 33.72±9.77) were respectively shown in the Table 1. There were no statistical differences in both age and gender among three groups.

**Table 1:** Clinical characteristics of participants among groups.

**Characteristics**		**I (*n* = 28)**	**II (*n* = 47)**	**III (*n* = 27)**	**Z/F/c^2^**	***P***
Age (Year)		31.79 ± 12.83	38.11 ± 13.16	33.72 ± 9.77	2.63	0.08
Onset Age (Year)		22.39 ± 8.82	33.98 ± 12.87	/	−3.82	<0.001
Gender	M	14	20	17	3.46	0.15
	F	14	27	10		
Education level (Year)		13.32 ± 3.37	11.26 ± 2.86	13.59 ± 3.30	6.43	<0.001
Scores of HAMD		31.04 ± 7.92	30.06 ± 7.45	/	−0.53	0.59
Scores of HAMA		13.86 ± 6.01	15.39 ± 6.01	/	1.07	0.29
Attack Frequency		4.64 ± 2.91	1.85 ± 1.72	/	−5.10	<0.001
Illness Course (Month)		109.79 ± 126.93	55.66 ± 98.20	/	−3.81	<0.001
Psychotropic medications, n		23	25			
Antidepressants, n		6	25			
Lithium, n		7	0			
Antiepileptic, n		15	2			
Anxiolytics, n		4	5			
Antipsychotics, n		12	2			
Medication-free		5	22			

*I, bipolar depression group; II, unipolar depression disorder group; III, healthy control group. Chi-square test, gender; ANOVA, age, education level; T-test, scores of HAMD, scores of HAMA, onset age, attack frequency, illness course*.

### Differences of mfALFF values among groups

**Figure 1:**
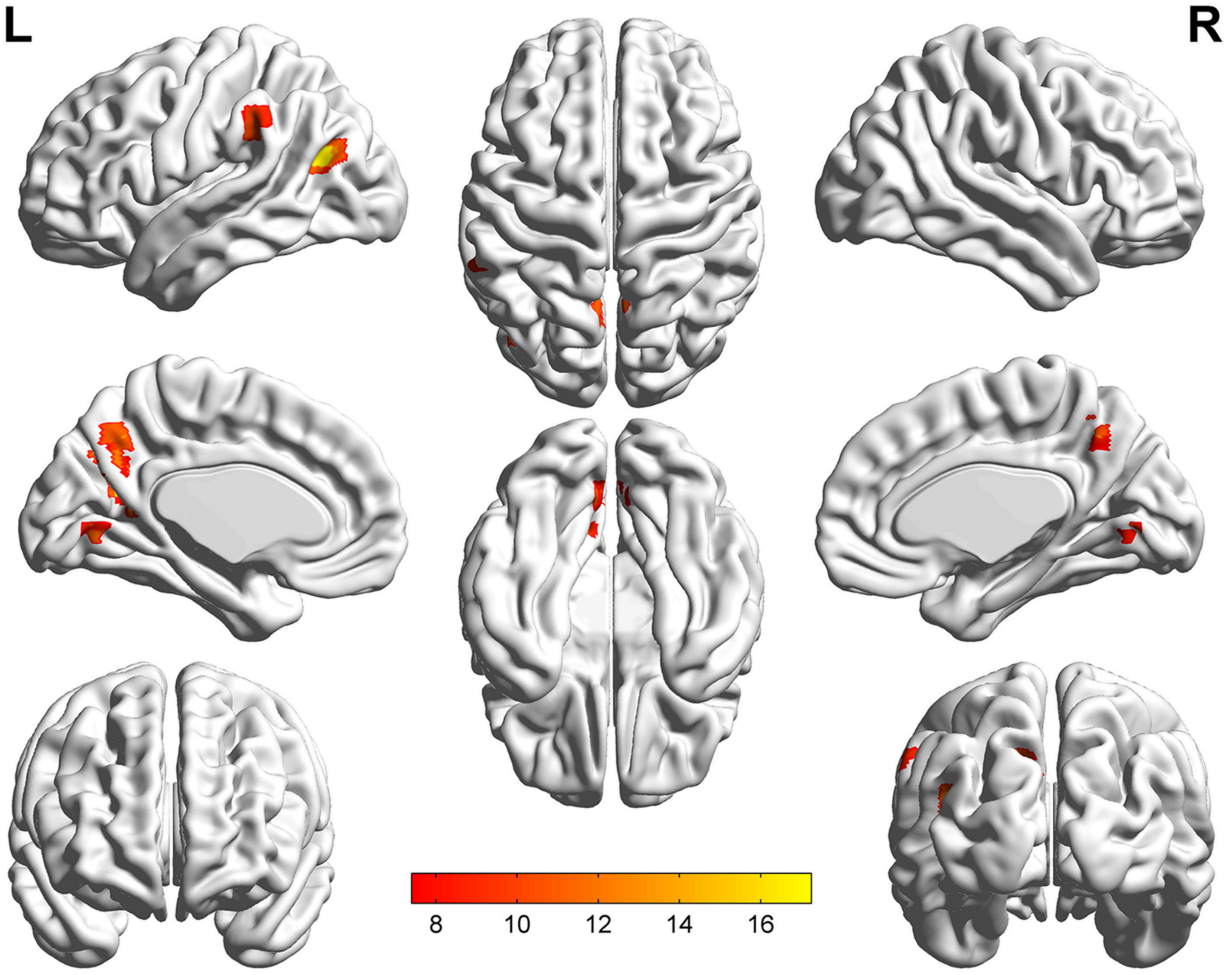
Statistical maps showing ANOVA results of mfALFF value among patients with UD, BD, and HCs. The different regions mainly including precuneus, the left MTG, the left IPL, and lingual gyrus. Threshold was set at voxel-wise *P* < 0.001 (uncorrected), with a cluster size >10 voxels. The resulting images are overlaid on the MNI1152 template. The color bars represent the range of *F*-value.

The significant differences of mfALFF values as the result of ANOVA among UD, BD, and healthy control groups were shown in Figure 1. The anatomical location of brain regions and intensity of activity were described in Table 2. The brain regions with significant differences were located in precuneus, the left MTG, the left IPL, and lingual gyrus.

**Table 2:** Brain regions with significant differences of mfALFF values in ANCOVA among UD, BD, and HC groups.

**Brain regions**	**L/R**	**Cluster size (voxels)**	**BA**	**MNI Coordinate (mm)**			***F***
				**x**	**y**	**z**	
Precuneus	L	40	7	−18	−57	21	16.62
				−12	−63	36	16.37
	/	15	7	0	−57	45	14.11
MTG	L	15	39	−39	−66	21	16.60
IPL	L	11	40	−57	−36	33	10.50
Lingual gyrus	/	11	18	0	−69	0	11.77

*ANCOVA, analysis of covariance; BA, brodmann area; MNI, Montreal Neurological Institute; HC, healthy control; L, Left; R, Right*.

**Figure 2:**
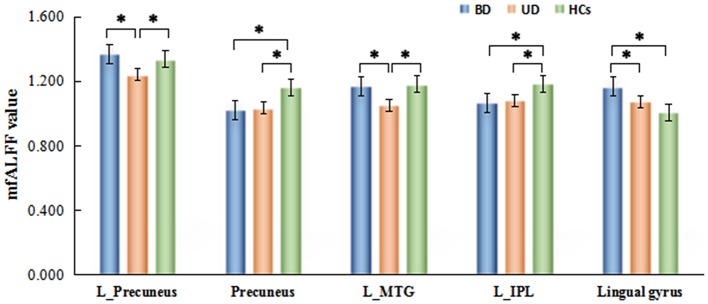
Brain regions with significant differences of mfALFF values inter-groups. ^*^Means *p* < 0.001.

By a *post-hoc* test, BD group showed a significant increase of mfALFF value in lingual gyrus (*p* = 0.000), but significant decrease of mfALFF value in precuneus (*p* = 0.000), and the left IPL (*p* = 0.000) vs. healthy control group. Relative to the control group, a significant decreased mfALFF values were observed in the left precuneus (*p* = 0.001), precuneus (*p* = 0.000), the left MTG (*p* = 0.000), and the left IPL (*p* = 0.000) in UD group. Remarkable increased of mfALFF values in the left precuneus (*p* = 0.000), the left MTG (*p* = 0.000), and lingual gyrus (*p* = 0.004) were observed in BD group in contrast to UD group. Details are shown in Table 3 and Figure 2.

**Table 3:** Brain regions with significant differences of mfALFF values inter-groups.

**Brain regions**	**BD**	**UD**	**HC**	***P*****-value**		
				**UD vs. BD**	**UD vs. N**	**BD vs. N**
L_Precuneus	1.370 ± 0.124	1.243 ± 0.090	1.336 ± 0.104	0.000^*^	0.001^*^	0.666
Precuneus	1.023 ± 0.063	1.034 ± 0.078	1.165 ± 0.106	1.000	0.000^*^	0.000^*^
L_MTG	1.170 ± 0.121	1.052 ± 0.081	1.182 ± 0.088	0.000^*^	0.000^*^	1.000
L_IPL	1.068 ± 0.088	1.083 ± 0.079	1.187 ± 0.081	1.000	0.000^*^	0.000^*^
Lingual gyrus	1.167 ± 0.137	1.075 ± 0.112	1.010 ± 0.106	0.004^*^	0.066	0.000^*^

* *Means p < 0.01*.

### Correlation analysis with the clinical characteristics

**Figure 3:**
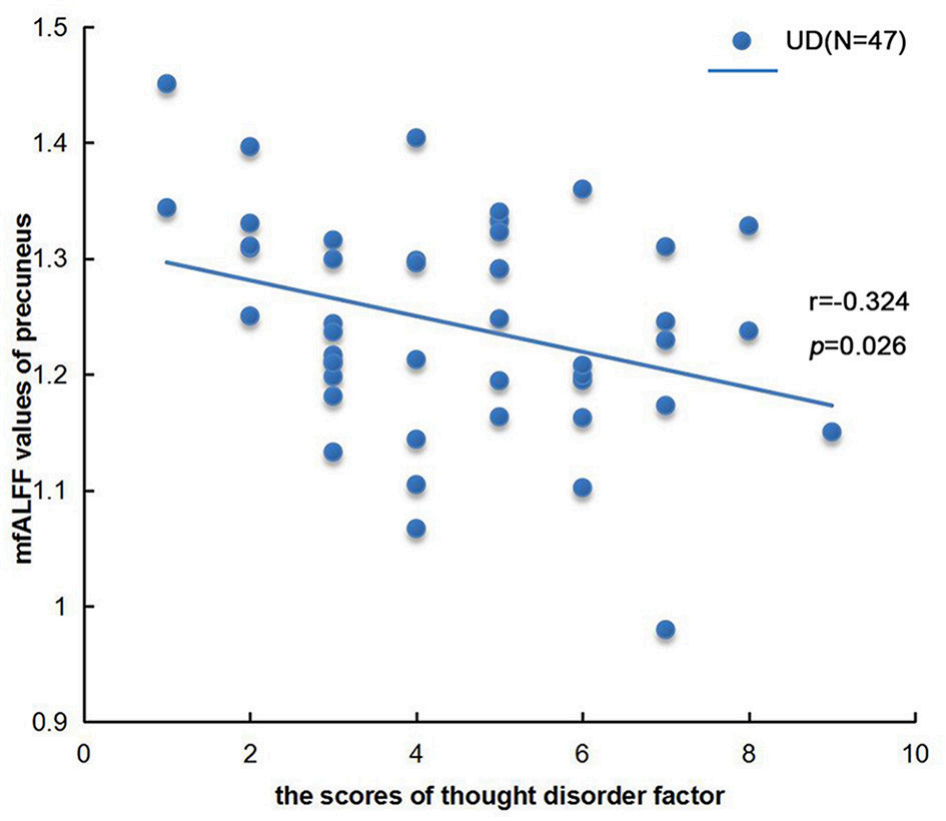
Correlation analysis between mfALFF values of precuneus in UD group and thought disorder factor (factor 3 of HAMD). *n* = 47, *p* < 0.05.

We found a negative correlation between the cognitive disorder (factor 3 of HAMD) and the mfALFF value of precuneus in UD group, as shown in Figure 3. There were not any other significant relationships between the different fALFF values in the left PCC, the left IPL and lingual gyrus with clinical variants such as onset age, illness course, scores of HAMD, and scores of HAMA as well.

## Discussion

In the current study, we examined the fALFF changes in patients with BD and UD compared with healthy controls by using resting-state fMRI. The results revealed that fALFF values noticeably varied in precuneus, the left MTG, the left IPL, and lingual gyrus among BD, UD, and HC group. These altered values mainly occurred in DMN (excluding lingual gyrus) in the UD patients. The results also showed that the score of thought disorder factor in patients with UD was negatively correlated with the fALFF values in the precuneus. However, we noticed the altered neural activity of lingual gyrus, precuneus and the left IPL in patients with BD in comparison with HC group. Meanwhile, BD patients showed significantly increased fALFF values in the left precuneus, the left MGT and lingual gyrus relative to UD patients.

This research indicated that the decreased intrinsic activity located in part of DMN including precuneus, the left MGT and left IPL in patients with UD relative to HCs. The DMN is composed of cerebral regions which are activated when the brain is wakefully rest, but are deactivated during the goal-oriented tasks (36). DMN is thought to be related to cognitive function of self-awareness, episodic memory, salience, and interactive modulation (37, 38). Previous neuroimaging studies have highlighted the involvement of DMN in the pathophysiology of UD by index of functional connectivity, effective connectivity, Reho, and fALFF (39). Among these different analysis methods, the fALFF analysis allows us to directly detect regional signal changes of spontaneous activity at rest (24). Our results also highlighted the importance of fALFF analysis in reflecting intrinsic neural activity within specific regions inside of the DMN.

In present study, subjects with UD showed decreased spontaneous neural activity of the left MTG compared to both subjects with BD and HC participants. As a part of DMN, the lateral temporal regions are considered to process sentence comprehension and language (40–42), especial the retrieval of lexical-syntactic information from memory (43). Our results were in line with previous findings. For example, Guo et al found decreased ALFF values in the left MTG (44). Meanwhile, decreased fALFF values in the left MTG was observed in first-episode, treatment-naïve patients with major depressive disorder (30, 45). Evidences from task-fMRI studies also supported the abnormality of the MTG in UD patients, as patients exhibited decreased activation in the left MTG during sad facial expression recognition (46). On the other hand, one study found that, compared to UD patients, BD patients showed decreased activation in the MTGduring emotional processing task (47). Thus, the finding of MTG in this study might be part of neural underpinning for the different clinical characteristics in depressive state between UD and BD patients.

Precuneus, serving as a core hub of the DMN, may be related to self-referential processing and mental representations (48). Similar to previous studies (19, 45, 49), our study reported decreased fALFF value of precuneus in UD patient and BD patients. We also found that there existed a drastic negative correlation between the fALFF values of precuneus and the score of cognitive disorder. A meta-analysis reported the decreased brain activity in posterior precuneus in first-episode, drug-naïve UD patients (50), while Zhu et al. found that the decreased functional connectivity between precuneus and other regions of posterior medial cortex was negatively correlated with the overgeneral autobiographical memory (AM) (51). Furthermore, undergoing the sad mood induction, patients with chronic UD showed the decreased functional connectivity between precuneus and PCC (52). In our study, we found that the fALFF values of precuneus in BD group were significantly higher than UD group. Meanwhile, structural differences in precuneus have been found in comparisons between BD and UD patients (53). Young et al. also reported the decreased activity of precuneus in BD patients relative to UD patients during specific AM of negative memories (54). Therefore, it might be suggested that the abnormal spontaneous neural activity in precuneus may play an important role in AM (54), and maybe provide further evidence for distinguishing the differentiation of affective disorders.

The UD and BD group also showed decreased fALFF value in IPL compared to HC groups. Previous studies have found that IPL plays a critical role in emotional regulation, self-referential processing and response inhibition (55), while IPL is also observed to be related with monetary rewards (56). Several previous studies found the functional changes of IPL in patients with UD (30, 56–58). Guo et al. found that UD patients had decreased functional connectivity between the left cerebral lobule and the left IPL compared to healthy controls (57), which maybe related with the deficits of cognitive control in patients with UD (58). Our study also found decreased fALFF value in IPL of the patients with BD compared to HC. Previous studies (53, 59) indicated the alterations in cerebral blood flow or gray matter volume of IPL in BD might be related to the prominent deficits in response inhibition. Therefore, the further studies about IPL among the patients of UD and BD should be carried out in terms of the cognitive control function, which may present the similar symptom in depressive state.

Lingual gyrus was another region with significantly increased fALFF values in BD group, compared with both UD group and HC group. Lingual gyrus, as the key region of visual cognitive network, helps process the facial recognition and emotion recognition (60). Lingual gyrus also plays an important role in the integration of visual information and introspective stimuli. Xu et al. firstly found the decreased value of fALFF in lingual gyrus in patients with BD (61). The altered function of lingual gyrus in BD patients have been found to be associated with the deficits of visual sensory processing (62) and episodic memory (63). Taken together, our result indicates that the decrease of fALFF values in lingual gyrus might be the sensible index reflecting the visual cognitive dysfunction in BD.

The main limitation of the present study is uncontrolled effects of medication, especially that medication load was higher for individuals with BD than for those with UD, which might lead to type II errors (64), although our findings are similar to previous studies. Secondly, patients with BD in our study showed earlier age of onset, more depressive episodes and longer illness course relative to UD patients. These atypical characteristics of depressive episode are regarded as risk factors for bipolar disorder (65). Thirdly, the relatively small sample size would be a limitation for the interpreting of our potential findings. Although the sample size in our study is comparable to many previously published studies, larger-sized sample should hypothetically yield more representative results (66). However, one previous study also argued that the inter-subject correlation (ISC) statistics with 20 subjects had converged close to a large sample ISC statistics with 130 subjects averagely (67). Finally, our study failed to replicate the results in the other independent sample due to the limited sample size. Considering all the above mentioned, further work, combining clinical phenotype, and adopting longitudinal methods, is required to replicate our findings and to provide more conclusive evidences.

## Conclusion

In summary, patients with BD showed distinctive pattern of intrinsic activity in the lingual gyrus compared to UD patients and HCs, suggesting potential different pathophysiological mechanisms of BD. The similar fALFF changes located in the precuneus and the left IPL between BD and UD patients might be associated with the coexisted symptoms in depressive state. Furthermore, the significant aberrant spontaneous neural activity in left MTG and the left precuneus may provide potential neural underpinning to distinguish BD from UD. Therefore, aberrant brain intrinsic activity may highlight new perspectives on investigating neuroimaging-based biomarkers in future studies of BD vs. UD.

## Ethics statement

This study was carried out in accordance with the recommendations of “the Investigational Review Board (IRB00002733-Shanghai mental health center, China)” with written informed consent from all subjects. All subjects gave written informed consent in accordance with the Declaration of Helsinki. The protocol was approved by the “the Investigational Review Board (IRB00002733-Shanghai mental health center, China).”

## Author contributions

MQ, DP, and TS conceived and designed the experiments. MQ, DP, TS, JZ, HZ, and YH performed the experiments. MQ, DP, JS, and CW analyzed the data. MQ, DM, HZ, DP, and TS wrote the paper.

### Conflict of interest statement

The authors declare that the research was conducted in the absence of any commercial or financial relationships that could be construed as a potential conflict of interest. The reviewer JT and handling Editor declared their shared affiliation.
